# Comparison of Access to Primary Care Medical and Dental Appointments Between Simulated Patients Who Were Deaf and Patients Who Could Hear

**DOI:** 10.1001/jamanetworkopen.2020.32207

**Published:** 2021-01-21

**Authors:** Elizabeth Schniedewind, Ryan P. Lindsay, Steven Snow

**Affiliations:** 1 Kasiska Division of Health Sciences, Idaho State University, Meridian; 2 Idaho Council for the Deaf and Hard of Hearing, Boise

## Abstract

**Importance:**

Primary medical and dental clinics may accept fewer people who are deaf as patients than persons who can hear, and clinics may deny requests by patients who are deaf for American Sign Language (ASL) interpretation at appointments when necessary, creating diminished access to primary medical and dental care.

**Objective:**

To compare the rate at which patients who are deaf are offered primary care medical or dental appointments with the rate at which appointments are offered to patients who can hear in a real-world context.

**Design, Setting, and Participants:**

This cross-sectional study used a simulated patient (SP) call audit method. Simulated patients (4 who could hear and 4 who were deaf) followed a call script in which an adult sought to establish care, requesting new patient appointments from a statewide stratified random sample of clinicians listed in the Idaho Medical and Dental Associations member databases at 445 clinics (334 primary care and 111 general dentistry) throughout Idaho. Simulated patients who were deaf also requested interpreting services at the appointment. Calls were made between June 7 and December 6, 2018. Data analysis was conducted from December 2019 to April 2020.

**Exposures:**

Simulated patients who were deaf or could hear called primary care medical or dental clinics from the sampling frame and requested an appointment, supplying the same basic information. In addition, SPs who were deaf requested that an ASL interpreter be provided by the clinic for the appointment.

**Main Outcomes and Measures:**

Rates of new appointments offered and, for SPs who were deaf, whether ASL interpreter services were confirmed for the appointment.

**Results:**

Two male and 2 female SPs who could hear were successful on 210 occasions (64.4%) when requesting a new patient appointment compared with 2 male and 2 female SPs who were deaf who were successful on 161 occasions (49.1%) (*P* < .001). Simulated patients who could hear were nearly 2 times more likely to secure appointments than were SPs who were deaf (adjusted odds ratio, 1.88; 95% CI, 1.27-2.79). For SPs who were deaf, 80 unsuccessful appointment requests (48.2%) were associated with a request for interpretation.

**Conclusions and Relevance:**

The findings suggest that in a statewide representative sample, access to primary medical and dental care for patients who are deaf is significantly reduced. Patients who are deaf may not receive an appointment if they request interpreter services, even when such services are required to provide effective communication.

## Key Points

**Question:**

Are patients who can hear more likely to secure primary care medical or dental appointments than patients who are deaf?

**Findings:**

In this cross-sectional study including 8 simulated patients, those who could hear were nearly 2 times more likely to secure appointments at 445 clinics than were simulated patients who were deaf. Among 80 unsuccessful requests made by simulated patients who were deaf, 48% were associated with a request for interpretation.

**Meaning:**

The findings of this study suggest the patients who are deaf and request interpreter services may experience diminished access to care at primary medical and dental clinics.

## Teaser

This cross-sectional study compares the rate at which patients who are deaf are offered primary care medical or dental appointments with the rate at which appointments are offered to patients who can hear.

## Introduction

Effective communication between a health care professional and patient has been called the “heart and art of medicine.”^1^ Effective communication fosters an exchange of information between the clinician and patient,^2^ allows the clinician to ask open-ended questions,^1^ and improves treatment outcomes.^3^ In the case of patients who are deaf and communicate in American Sign Language (ASL), clinicians may be unable to engage in this dialogue successfully without the services of an ASL interpreter, who must be supplied if such services are necessary to ensure that communication with patients who are deaf is as effective as communication with those who can hear.^4,5^

Patients who are deaf have reported that the main communication barriers experienced in health care settings are the lack of ASL interpreters and the lack of use of sign language by health care professionals.^6^ Inadequate comprehension during health care encounters and the lack of engagement^7^ may contribute to feelings of fear, mistrust, and frustration^8^ or the avoidance of health care professionals altogether.^9^ Patients who are deaf have an increased likelihood of poor physician-patient communication and reduced satisfaction with care,^10^ may be unable to share important medical history or ask questions,^11^ or may be unable to establish a strong relationship with their primary care professionals.^12^ This lack of communication can result in misunderstanding of diagnoses^13^ and poor adherence to treatment regimens.^14^ Without interpreters, health care professionals who are not fluent in ASL may be unable to appreciate subtle presentations or symptoms of conditions that require communication for assessment.^15^

The provision of interpreter services for patients who are deaf is associated with better adherence to preventive screening recommendations.^16^ In one study, failure to provide requested interpreter services resulted in 82% of patients being unable to understand their diagnosis, 70% not understanding the guidelines for their treatment, and 63% choosing to discontinue care.^6^ The communication preference of 93% of patients who are deaf is to have either interpretation services at their appointment or a clinician who is language concordant and capable of communicating directly.^17^

The need for effective communication is not limited to the patient-clinician encounter. Patients who are deaf report difficulty making health care appointments,^13,14^ failure to receive requested help from clinic staff,^13^ and an inability to successfully contact clinics independently because of communication barriers.^14^ Front desk staff may engage in gatekeeping or discriminatory actions^18^ when responding to patient requests made over the phone. The discrimination may be based on patient names, accent cues, or, in the case of patients who are deaf, an unfamiliarity with interpreted phone calls. Patients who are deaf typically request health care appointments using the video relay service (VRS), a federally funded interpreting service in which the person who can hear uses spoken English over the traditional telephone and the deaf party uses ASL via videophone, which is then interpreted.^19^ The VRS calls often begin with an announcement by the interpreter that the call is from a person using sign language. Interaction with front desk staff can impact the rate of secured appointments^18^ and increase the burden patients bear to advocate for service.^20^

To examine barriers to primary medical and dental care and communication access from the perspective of patients who are deaf, we performed a cross-sectional audit study that examined differences in the rate of new patient appointments secured by simulated patients who were deaf and patients who could hear as the primary study objective. Secondary objectives included an exploration of the reasons that appointment requests from SPs who were deaf were unsuccessful and identification of clinic personnel present or time points in the process of securing an appointment in which a denial was more likely to occur. We hypothesized that SPs who were deaf would be offered fewer new patient appointments and that requests for interpreter services would, in some cases, cause an initially successful request for an appointment to fail at a later time.

## Methods

### Design and Setting

Idaho State University, the Idaho Council for the Deaf and Hard of Hearing, and Deaf community members from Boise, Idaho, formed a community-based participatory research collaboration. Identification of current strategies to access care, concerns, and research focus preferences were documented in focus group meetings conducted exclusively in ASL. The Idaho State University Institutional Review Board determined that our study was not human subjects research and so was exempt from the need for approval. This study complied with the Strengthening the Reporting of Observational Studies in Epidemiology (STROBE) reporting guideline. Data linking providers and clinic addresses to assigned clinic identifications have been destroyed.

**Figure 1.**
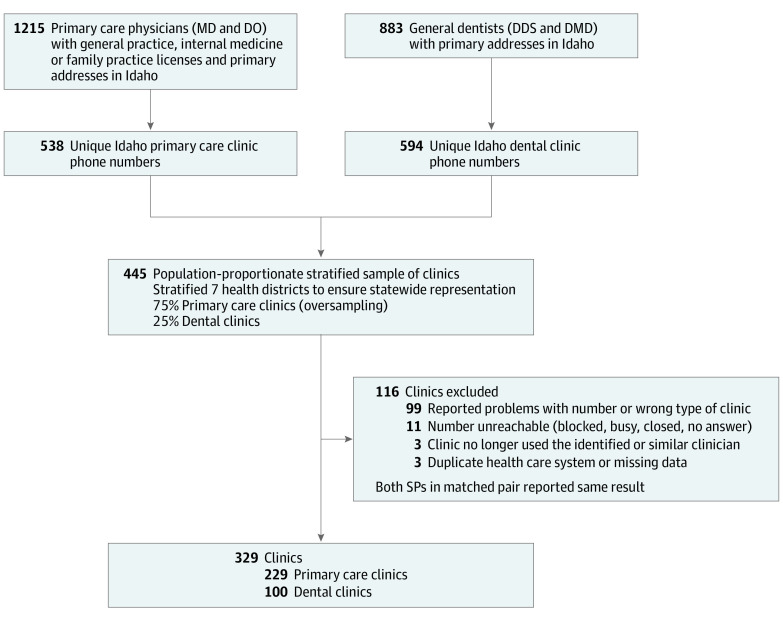
Sampling Procedure and Reasons for Clinic Exclusion DDS indicates doctor of dental surgery; DMD, doctor of dental medicine; DO, doctor of osteopathy; MD, medical doctor.

A sampling frame was created from board certification lists of primary care and general dentistry health care professionals that were matched to 2098 clinics and hospitals statewide using Google geolocation application programming interface. Of these primary care (1215 [57.9%]) and general dentistry (883 [42.1%]) clinics in Idaho, 1132 clinics (53.9%) possessed unique clinic phone numbers. A population-proportionate stratified sample that represented 7 health districts was determined, and 445 clinics (39.3%) comprising 75% primary care (n = 334, oversampling) and 25% (n = 111) general dentistry clinics were eligible for study inclusion. Each clinic included in the sample was called before the study to verify that the number was in service. A total of 116 clinics were excluded, resulting in a final sample of 329 (primary care, 229; dental, 100) clinics (Figure 1).

Two male and 2 female simulated patients (SPs) who could hear were selected, as were 2 male and 2 female SPs who were deaf to serve as matched SPs seeking new patient appointments to establish care after recently moving to the area. The audio and/or video were recorded for each call to ensure study protocol adherence and verification of the call outcome. Simulated patients were trained in the use of a standardized script and completed test calls with supervision to ensure consistency. Simulated patients self-selected fictitious names and were assigned individual local phone numbers with message capability for follow-up. Scripts included insurance plan information (Blue Cross of Idaho, state employee plan) and fictitious local addresses in each health district. If SPs were unable to provide answers to requests for specific information, they explained that they had recently relocated. Simulated patients requested appointments with the randomly selected clinician, but if that clinician was not accepting new patients or was unavailable within the limits set by the study, SPs asked if another clinician was available, including physician assistants and nurse practitioners.

Simulated patients who were deaf received additional training from the Idaho Council for the Deaf and Hard of Hearing and Idaho State University in study protocols specific to the request for interpreter services. Focus group members selected the VRS provider to be used, the type and amount of self-advocacy included in the call script, and contact information for an interpreter referral service that SPs gave to clinic staff upon request or as a suggestion of a resource to call when securing interpreters. Simulated patients who were deaf verified that the perceived sex of the VRS interpreter was consistent with their own.

To minimize inconvenience and prevent unnecessary charges, study protocol dictated that only appointments offered at least 4 weeks after the initial call was made were accepted. Simulated patients called clinics again to cancel appointments with at least 2 weeks’ notice to prevent charges for interpreter services and/or loss of clinician availability for patient visits. An interpreter referral service assisted with the study by identifying appointment requests for SPs who were deaf using the fictitious names, providing a confirmation of services to the clinic, and ensuring that no charges were incurred by clinics as a result of the request for interpreter services.

Appointment requests were considered successful for SPs who could hear were if they were given an appointment date/time. The requests of SPs who were deaf were considered successful if they received an appointment date/time and confirmation of interpreting services at the appointment. Population density,^21^ perceived sex of the VRS interpreter, clinic type, and region were the factors examined in association with successfully securing a new patient appointment. A subanalysis included reasons that appointments were not secured by SPs who were deaf regarding interpreter services and factors associated with that outcome.

### Statistical Analysis

Data analysis was conducted from December 2019 to April 2020. Differences in descriptive statistics using Pearson χ^2^ and 2-tailed, unpaired *t* tests were performed for SPs securing new patient appointments compared with those who did not and among SPs who were deaf who were unsuccessful in securing a new patient appointment because of an interpreter-related reason compared with all other reasons. Using conditional fixed-effects logistic regression in Stata, version 13 (StataCorp LLC), we described the likelihood of securing a new patient appointment among SPs who were deaf compared with the matched SPs who could hear. Simulated patients were matched 1:1 by clinic identification number. Among SPs who were deaf, bivariate and multivariable logistic regression were used to assess demographic and call-related factors associated with having an interpreter-related reason for an unsuccessful attempt at securing an appointment. Collinearity was assessed using tolerance values less than 0.1. The statistical significance threshold was α = .05.

## Results

There were 1096 call records completed by SPs between June 7 and December 6, 2018. Unsuccessful appointment requests fell into 3 categories: protocol requirements of either the sampled clinic or study protocol, failure to meet clinic screening requirements, and interpreter-related denials. Requests were denied because clinicians were not accepting new patients on 23 occasions (19.8%) for SPs who could hear and 31 occasions (18.6%) for those who were deaf. For 4 unsuccessful appointment requests (2.4%), SPs who were deaf were told that the clinic did not accept their insurance, which did not occur with SPs who could hear.

**Figure 2.**
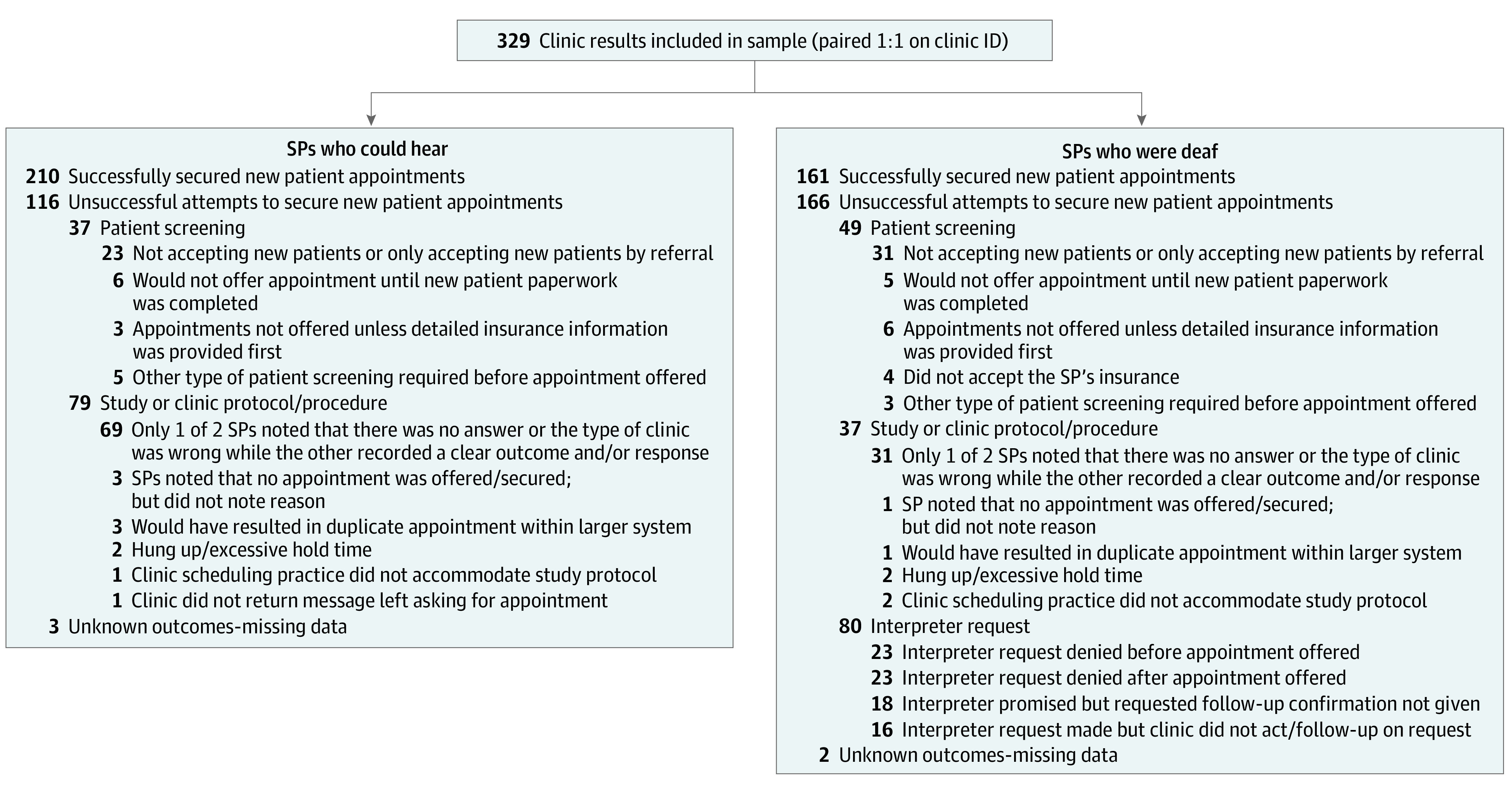
Outcomes of New Patient Appointment Requests ID indicates identification; SP, simulated patient.

Four types of interpreter-related denials were identified: (1) the request for interpreter services was denied before the appointment date/time was offered (23 [28.75%]); (2) the request for interpreter services was denied after the appointment date/time was offered (23 [28.75%]); (3) the appointment date/time and interpreter request were approved, but no confirmation of the interpreter services was given, despite follow-up attempts (18 [22.5%]); and (4) de facto denials, which occurred when the SP who was deaf requested interpreter services but was not offered an appointment (16 [20.0%]). On occasions of de facto denials, clinic staff said that they would call back with an approval or denial of the request but failed to do so after 2 follow-up calls were made (Figure 2).

Approximately half of SPs who were deaf were able to secure a new patient appointment (161 [49.1%]) compared with 210 (64.4%) among SPs who could hear (*P* < .001) (Table 1). Attempts to secure appointments were more likely to be successful at dental clinics (63.5%) compared with primary care clinics (53.7%) (*P* < .001). In terms of odds of success, SPs who could hear were nearly 2 times more likely to secure new patient appointments compared with SPs who were deaf (adjusted odds ratio [aOR], 1.88; 95% CI, 1.27-2.79), after controlling for the number of contacts made and the sex of the SP.

**Table 1.** Demographic and Call-Related Factors Overall and by Success Status

Characteristic	No. (%)			*P* value^a^	Unsuccessful attempts, OR (95% CI)^b^	
	Overall (n = 654)	Success				
		Yes (n = 371)	No (n = 283)		Unadjusted	Adjusted
Simulated patients						
Not deaf	326 (49.9)	210 (64.4)	116 (35.6)	<.001	1 [Reference]	1 [Reference]
Deaf	328 (50.1)	161 (49.1)	167 (50.9)		2.09 (1.47-2.97)	1.88 (1.27-2.79)
Demographics						
Clinic type					Matched	Matched
Primary care	454 (69.4)	244 (53.7)	210 (46.3)	.02		
Dental	200 (30.6)	127 (63.5)	73 (36.5)			
Density					Matched	Matched
Urban (metro MSA)	468 (71.6)	255 (54.5)	213 (45.5)	.07		
Rural (micro MSA or neither)	186 (28.4)	116 (62.4)	70 (37.6)			
Region					Matched	Matched
North	143 (21.9)	81 (56.6)	62 (43.4)	.53		
Southwest	293 (44.8)	160 (54.6)	133 (45.4)			
Southeast	218 (33.3)	130 (59.6)	88 (40.4)			
Sex						
Male	320 (49.6)	184 (57.5)	136 (42.5)	.70	1 [Reference]	1 [Reference]
Female	334 (50.4)	187 (56.0)	147 (44.0)		1.00 (0.63-1.58)	1.20 (0.71-2.01)
Call related						
No. of contacts, mean (SD)	x̅ = 1.2 (0.57)	x̅ = 1.2 (0.58)	x̅ = 1.3 (0.56)	.57	1.73 (1.13-2.66)	1.31 (1.27-2.79)

Abbreviations: MSA, metropolitan statistical area; OR, odds ratio.

^a^ Significance determined using Pearson χ^2^ analysis or *t* test.

^b^ Conditional fixed-effects logistic regression model of unsuccessful attempts (648 or 324 matched pairs).

Simulated patients who were deaf documented reasons given by clinic staff when an appointment request was unsuccessful; the denial was associated with an interpreter request in 80 of 166 (48.2%) requests. Among these denials, attempts to secure appointments at dental clinics were more likely than at primary care clinics to have an interpreter-related reason for not securing the appointment (*P* < .001) (Table 2). The mean (SD) number of appointment requests made by SPs who were deaf that had an interpreter-related reason for an unsuccessful attempt was 1.6 (0.78) contacts compared with 1.2 (0.49) contacts for those unsuccessful for other reasons (*P* < .001). Demographic factors independently and positively associated with not securing an appointment for interpreter-related reasons were being a dental clinic compared with primary care clinic (aOR, 6.86; 95% CI, 3.01-15.64), women compared with men (aOR, 2.41; 95% CI, 1.14-5.12), southeast region compared with southwest region of Idaho (aOR, 2.59; 95% CI, 1.08-6.26), and the number of times the SP who was deaf had contact with the clinic (aOR, 2.46; 95% CI, 1.29-4.69) (eTable in the Supplement).

**Table 2.** Demographic and Call-Related Factors Associated With Having an Interpreter-Related Reason for Unsuccessful Attempt at Accessing Health Care Among 166 Deaf Simulated Patients

Characteristic	Interpreter-related reason for unsuccessful attempt among deaf simulated patients, No. (%)		*P* value^a^	Odds ratio (95% CI)	
	Yes	No		Unadjusted	Adjusted
Clinic type					
Primary care (n = 111)	38 (34.2)	73 (65.8)	.001	1 [Reference]	1 [Reference]
Dental (n = 55)	42 (76.4)	13 (23.6)		6.21 (2.98-12.95)	6.86 (3.01-15.64)
Density					
Urban (metro MSA) (n = 122)	54 (44.3)	68 (55.7)	.09	1 [Reference]	1 [Reference]
Rural (micro MSA or neither) (n = 44)	26 (59.1)	18 (40.9)		1.82 (0.90-3.66)	1.83 (0.80-4.22)
Region					
North (n = 35)	16 (45.7)	19 (54.3)	.25	1.12 (0.50-2.51)	1.04 (0.40-2.68)
Southwest (n = 77)	33 (42.9)	44 (57.1)		1 [Reference]	1 [Reference]
Southeast (n = 54)	31 (57.4)	23 (42.6)		1.80 (0.89-3.63)	2.59 (1.08-6.26)
Sex					
Male (n = 85)	37 (43.5)	48 (56.5)	.22	1 [Reference]	1 [Reference]
Female (n = 81)	43 (53.1)	38 (46.9)		1.47 (0.80-2.71)	2.41 (1.14-5.12)
Call related					
No. of contacts	x̅ = 1.6 (s = 0.78)	x̅ = 1.2 (s = 0.49)	<.001	2.53 (1.46-4.40)	2.46 (1.29-4.69)

Abbreviation: MSA, metropolitan statistical area.

^a^ Pearson χ^2^ and *t* tests were used for individuals securing new patient appointments and among simulated patients who were deaf who were unsuccessful in securing a new patient appointment.

## Discussion

In this cross-sectional audit study of a statewide representative sample of primary medical care and general dentistry clinics, patients who are deaf experienced diminished access to care in both settings. Their requests to establish care were unsuccessful more frequently compared with requests made by patients who can hear. The requests SPs who were deaf made for appointments were unsuccessful largely owing to reasons associated with the need for interpreter services at the appointment. For SPs who were deaf, appointment requests were more likely to be unsuccessful at the point of interpreter-related requests if the patient was perceived to be female, from the southeast region of Idaho, or requesting an appointment at a primary care clinic, or if the patient had more contact with the clinic (eg, phone conversations and messages left by either party).

Access to basic health services is an important component of access to health care. Patients who have a usual health care professional, place, and source of care experience better health outcomes and are more likely to receive preventive services and screenings.^22^ Barriers that limit access to services likely contribute to health disparities, inadequate health literacy, and incomplete health knowledge among people who are deaf.^23^ The decreased number of new patient appointments secured by SPs in this study who were deaf underscores the challenge that patients who are deaf face when attempting to establish care with a health care professional. People who are deaf have been found to have fewer physician visits,^10^ be less likely to have visited a physician in the preceding 2 years,^24^ be more likely to access care in emergency department settings,^25^ and be more likely to avoid health care professionals owing to a lack of communication or lack of an interpreter.^6^

The Department of Justice states that sign language interpreters are generally needed for health care communication as common as “taking the medical history of a patient who uses sign language”^26^ and, as such, would have been necessary for effective communication at the appointments requested by SPs in this study who were deaf. When patients who are deaf do not have the full and equal use of the services and advantages offered by health care entities to patients who can hear, it is considered discrimination, according to the Americans with Disabilities Act.^21^ It is possible that the clinic staff did not make the request for interpreter services known to the health care professional, or neither the staff nor clinician was aware of the obligation to provide auxiliary aids and services, including interpreter services, necessary to achieve effective communication. The clinic staff or clinician may have believed that it was not their responsibility to pay for the cost of interpreter services. Simulated patients who were deaf at times received direction from clinic staff to bring a family member or friend to interpret for their appointment, which, unless there is an imminent threat to safety or welfare, does not comply with the Americans with Disabilities Act.^26^

Although it has been demonstrated that health care professionals vary in their understanding of their legal responsibilities to patients with disabilities,^27^ clinic staff may have engaged in explicit gatekeeping or discriminatory treatment of the SPs who were deaf. The VRS interpreters do not follow a scripted greeting, and some interpreters may have informed staff that a person who was deaf was calling or the staff person was able to surmise this based on background noise present during the call, since most VRS calls are made from a call center and present differently. As in another study,^9^ SPs who were deaf in our study reported being hung up on frequently and calling back several times before their call was accepted.

The National Association of the Deaf provides a consumer fact sheet instructing patients who are deaf to inform health care professionals in advance about their need for interpreting services,^4^ but this may not be successful. Nearly one-third of persons with hearing loss who participated in a survey reported that no arrangements were made to improve communication in health care environments, despite the fact that 93% of the respondents informed the health care professionals about their hearing loss.^28^ In our study, the number of appointments in which interpreter services would be provided may have been overestimated, because we considered a confirmation of services booked by clinic personnel to guarantee interpreter services would be provided at the appointment. However, this is not consistent with the findings of a previous study that indicated interpreter services were frequently promised to patients who were deaf but were not provided at the appointment.^29^

It has been suggested that, although communication problems are the most significant factor affecting access to health care services for patients who are deaf, these patients need to increase their demands for access.^30^ Training has been provided to deaf community members to self-advocate for accommodations in health care and other settings,^31^ yet the more contacts the SPs who were deaf had with the clinic, the less likely they were to receive a new patient appointment. This suggests that training clinic staff to respond appropriately to requests for accommodations might be a more successful approach.

Dental clinics were more than 6 times more likely to deny a new patient request for an interpreter-related reason than primary care clinics. In Idaho, the Medicaid dental plan is administered by Managed Care of North America Dental. In their participant manual, it is affirmed that they will arrange interpreter services for either a patient or a parent or guardian of a patient at no charge.^32^ Clinic staff may have denied the request for interpreter services because they were accustomed to a dental plan administrator providing these services. A new patient appointment at a dental clinic typically requires a comprehensive examination, including history and a treatment plan.^33^ The amount of communication required at a typical first appointment at a dental clinic varies significantly from that at subsequent appointments, and clinic staff may not have recognized the need for interpreter services and therefore denied the request. Furthermore, dental clinics, unlike primary care clinics, are less likely to be affiliated with a health care system. Primary care clinics affiliated with a health care system may have access to interpreter services through the parent organization.

### Limitations

Study limitations include the large number of clinics that were excluded for reasons such as an incorrect or disconnected phone number, not providing primary care or general dentistry services, no answer, or being closed. The interaction with front-desk and clinic administration staff may not be an accurate reflection of the clinician’s preference, but the interaction might accurately reflect the experience of patients who are deaf. The ability to generalize findings from this single-state study may be limited or enhanced owing to the proportion of population-representative rural regions in our study.

## Conclusions

To our knowledge, this study is the first report supported by empirical data of the difficulties experienced by patients who are deaf who request interpreter services at health care appointments. In this statewide representative sample, access to primary and dental care for patients who are deaf was significantly reduced. A request for interpreting services, even when required for effective communication, was the most common reason appointment requests by patients who are deaf were unsuccessful. Training of clinic staff may result in improved access to health care for patients who are deaf.

## Notes

Supplement. **eTable.** Study Data by Office ID
