# Supplementary material for: Comparison of Access to Primary Care Medical and Dental Appointments Between Simulated Patients Who Were Deaf and Patients Who Could Hear
Source: JAMA Netw Open. 2021 Jan 21;4(1):e2032207. doi: 10.1001/jamanetworkopen.2020.32207 (PMC7821033; doi:10.1001/jamanetworkopen.2020.32207)
Supplement: Supplement. — eTable. Study Data by Office ID [file jamanetwopen-e2032207-s001.pdf]

Supplementary Online Content

Schniedewind E, Lindsay RP, Snow S. Comparison of access to primary care medical and dental appointments between simulated patients who were deaf and patients who could hear. *JAMA Netw Open*. 2021;4(1):e2032207. doi:10.1001/jamanetworkopen.2020.32207

eTable. Study Data by Office ID

This supplementary material has been provided by the authors to give readers additional information about their work.

© 2021 Schniedewind E et al. *JAMA Network Open.*

eTable. Study Data by Office ID

Office ID	Clinic type (0=Primary Care, 1=Dental)	Appointment Success (0=Yes, 1=No)	Gender (0=Male, 1=Female)	Simulated Patient (0=Not Deaf, 1=Deaf)	Region (1=Southwest, 2=North, 3=Southeast)	Density (0=Urban, 1=Rural)	Interpreter-related reason for unsuccessful attempt (0=No, 1=Yes, .=Missing)	Number of contacts
2	0	0	1	0	3	1		1
2	0	0	1	1	3	1		2
3	1	1	1	1	1	0	1	1
3	1	0	1	0	1	0		2
4	0	0	1	1	1	1		1
4	0	1	1	0	1	1	0	1
5	0	0	1	0	1	0		2
5	0	1	1	1	1	0	1	2
6	0		1	1	3	0	0	1
6	0	0	1	0	3	0		1
7	1	1	1	1	2	0	1	2
7	1	0	1	0	2	0		2
8	1	1	1	1	1	0	1	2
8	1	0	1	0	1	0		1
10	0	0	1	0	3	1		1
10	0	0	1	1	3	1		1
11	0	0	1	0	3	0		1
11	0	0	1	1	3	0		1
12	0	1	1	1	1	1	1	2
12	0	0	1	0	1	1		1
13	0	1	1	1	3	0	1	2
13	0	1	1	0	3	0	0	1

© 2021 Schniedewind E et al. *JAMA Network Open.*

Office ID	Clinic type (0=Primary Care, 1=Dental)	Appointment Success (0=Yes, 1=No)	Gender (0=Male, 1=Female)	Simulated Patient (0=Not Deaf, 1=Deaf)	Region (1=Southwest, 2=North, 3=Southeast)	Density (0=Urban, 1=Rural)	Interpreter-related reason for unsuccessful attempt (0=No, 1=Yes, .=Missing)	Number of contacts
14	0	0	1	1	3	1		1
14	0	0	1	0	3	1		1
15	1	0	0	1	2	0		3
15	1	0	1	0	2	0		1
16	0	0	1	0	2	0		1
16	0	1	1	1	2	0	1	1
17	0	1	1	1	3	1	1	3
17	0	1	1	0	3	1	0	2
18	0	1	1	1	2	1	0	2
18	0	0	1	0	2	1		1
19	1	1	1	1	1	0	1	1
19	1	0	1	0	1	0		1
20	1	1	1	1	3	0	1	1
20	1	0	1	0	3	0		1
21	1	0	1	1	3	1		1
21	1	1	1	0	3	1	0	1
22	1	1	1	1	2	0	1	1
22	1	0	1	0	2	0		1
23	1	0	1	1	2	0		1
23	1	0	1	0	2	0		1
24	0	0	1	1	3	1		1
24	0	0	1	0	3	1		1
25	1	0	1	1	3	1		1
25	1	0	1	0	3	1		1
26	0	1	1	1	1	1	0	1

© 2021 Schniedewind E et al. *JAMA Network Open.*

Office ID	Clinic type (0=Primary Care, 1=Dental)	Appointment Success (0=Yes, 1=No)	Gender (0=Male, 1=Female)	Simulated Patient (0=Not Deaf, 1=Deaf)	Region (1=Southwest, 2=North, 3=Southeast)	Density (0=Urban, 1=Rural)	Interpreter-related reason for unsuccessful attempt (0=No, 1=Yes, .=Missing)	Number of contacts
26	0	1	1	0	1	1	0	2
27	0	0	1	1	3	1		1
27	0	0	1	0	3	1		1
28	0	0	1	0	1	0		1
28	0	1	1	1	1	0	1	1
29	1	0	1	0	1	0		1
29	1	0	1	1	1	0		1
30	0	1	1	1	1	0	0	1
30	0	1	1	0	1	0	0	1
31	0	0	1	0	2	0		1
31	0	0	1	1	2	0		1
32	0	1	1	0	1	0	0	1
32	0	0	1	1	1	0		2
33	0	1	1	0	2	0	0	1
33	0	0	1	1	2	0		1
34	0	0	1	1	1	1		1
34	0	1	1	0	1	1	0	1
35	0	0	1	1	1	0		1
35	0	0	1	0	1	0		1
36	0	0	1	0	1	1		1
36	0	0	1	1	1	1		1
37	0	0	1	1	3	1		1
37	0	0	1	0	3	1		2
38	1	1	1	1	1	0	1	2
38	1	0	1	0	1	0		1

© 2021 Schniedewind E et al. *JAMA Network Open.*

Office ID	Clinic type (0=Primary Care, 1=Dental)	Appointment Success (0=Yes, 1=No)	Gender (0=Male, 1=Female)	Simulated Patient (0=Not Deaf, 1=Deaf)	Region (1=Southwest, 2=North, 3=Southeast)	Density (0=Urban, 1=Rural)	Interpreter-related reason for unsuccessful attempt (0=No, 1=Yes, .=Missing)	Number of contacts
40	0	0	0	1	1	0		1
40	0	0	1	0	1	0		1
41	0	0	1	0	1	1		1
41	0	0	1	1	1	1		2
42	0	0	1	1	3	0		1
42	0	0	1	0	3	0		1
43	0	0	1	1	3	0		1
43	0	0	1	0	3	0		1
45	0	0	1	0	1	0		1
45	0	0	1	1	1	0		1
46	0	1	1	0	1	0	0	2
46	0	1	0	1	1	0	0	2
47	0	1	1	1	1	0	0	2
47	0	1	1	0	1	0	0	1
48	1	0	1	1	2	0		2
48	1	0	1	0	2	0		1
49	0	0	1	0	1	0		1
49	0	0	1	1	1	0		1
52	0	0	1	1	1	1		1
52	0	0	1	0	1	1		1
53	0	0	1	1	2	0		1
53	0	1	1	0	2	0	0	1
56	0	0	1	0	3	1		1
56	0	0	1	1	3	1		1
58	0	0	1	1	1	0		1

© 2021 Schniedewind E et al. *JAMA Network Open.*

Office ID	Clinic type (0=Primary Care, 1=Dental)	Appointment Success (0=Yes, 1=No)	Gender (0=Male, 1=Female)	Simulated Patient (0=Not Deaf, 1=Deaf)	Region (1=Southwest, 2=North, 3=Southeast)	Density (0=Urban, 1=Rural)	Interpreter-related reason for unsuccessful attempt (0=No, 1=Yes, .=Missing)	Number of contacts
58	0	1	1	0	1	0	0	1
59	1	0	0	0	1	0		1
59	1	1	1	1	1	0	0	1
60	0	0	1	1	1	0		1
60	0	0	1	0	1	0		1
61	0	1	1	1	1	1	1	2
61	0	0	1	0	1	1		2
62	0	0	1	1	3	0		2
62	0	0	1	0	3	0		1
63	1	0	1	0	2	0		2
63	1	0	1	1	2	0		2
65	0	0	1	1	1	0		1
65	0	1	1	0	1	0	0	1
66	0	1	1	0	1	0	0	1
66	0	0	1	1	1	0		1
67	0	1	1	0	1	0	0	1
67	0	1	1	1	1	0	0	1
68	0	1	1	0	1	0	0	1
68	0	1	1	1	1	0	0	1
70	0	0	1	1	3	1		2
70	0	0	1	0	3	1		1
72	1	0	1	1	1	0		1
72	1	0	1	0	1	0		1
73	0	0	1	0	2	1		1
73	0	1	1	1	2	1	1	1

© 2021 Schniedewind E et al. *JAMA Network Open.*

Office ID	Clinic type (0=Primary Care, 1=Dental)	Appointment Success (0=Yes, 1=No)	Gender (0=Male, 1=Female)	Simulated Patient (0=Not Deaf, 1=Deaf)	Region (1=Southwest, 2=North, 3=Southeast)	Density (0=Urban, 1=Rural)	Interpreter-related reason for unsuccessful attempt (0=No, 1=Yes, .=Missing)	Number of contacts
74	0	0	1	0	2	0		1
74	0	1	1	1	2	0	0	1
75	0	0	1	0	1	1		1
75	0	1	1	1	1	1	1	1
77	1	0	1	0	3	1		1
77	1	1	1	1	3	1	1	1
78	0	1	1	0	2	0	0	1
78	0	1	1	1	2	0	0	1
79	0	1	1	1	3	1	1	2
79	0	0	1	0	3	1		1
80	0	1	1	0	3	0	0	1
80	0	1	1	1	3	0	1	1
81	0	1	1	0	1	0	0	1
81	0	1	1	1	1	0	0	1
82	1	0	1	0	1	0		1
82	1	1	1	1	1	0	1	2
83	0	0	1	0	1	0		1
83	0	0	1	1	1	0		2
84	0	1	1	1	2	0	1	1
84	0	1	1	0	2	0	0	2
85	0	1	1	1	3	1	1	1
85	0	0	1	0	3	1		1
86	1	0	1	0	3	1		
86	1	0	1	1	3	1		
88	1	0	1	1	2	0		1

© 2021 Schniedewind E et al. *JAMA Network Open.*

Office ID	Clinic type (0=Primary Care, 1=Dental)	Appointment Success (0=Yes, 1=No)	Gender (0=Male, 1=Female)	Simulated Patient (0=Not Deaf, 1=Deaf)	Region (1=Southwest, 2=North, 3=Southeast)	Density (0=Urban, 1=Rural)	Interpreter-related reason for unsuccessful attempt (0=No, 1=Yes, .=Missing)	Number of contacts
88	1	0	1	0	2	0		1
89	0	0	1	1	2	0		1
89	0	0	1	0	2	0		1
90	0	0	1	0	1	0		2
90	0	0	1	1	1	0		1
92	0	1	1	1	1	0	1	1
92	0	1	1	0	1	0	0	1
93	0	1	1	1	1	0	0	1
93	0	1	1	0	1	0	0	1
94	1	1	1	0	1	0	0	2
94	1	0	1	1	1	0		1
95	0	1	1	0	3	1	0	2
95	0	1	1	1	3	1	0	1
97	0	0	1	1	2	0		1
97	0	0	1	0	2	0		2
98	1	0	1	0	3	1		1
98	1	1	1	1	3	1	1	2
99	0	0	1	0	3	1		1
99	0	1	1	1	3	1	1	1
101	0	0	1	0	1	0		1
101	0	0	1	1	1	0		1
102	1	1	1	1	1	0	1	2
102	1	0	1	0	1	0		1
103	1	1	0	1	1	0	0	1
103	1	0	1	0	1	0		1

© 2021 Schniedewind E et al. *JAMA Network Open.*

Office ID	Clinic type (0=Primary Care, 1=Dental)	Appointment Success (0=Yes, 1=No)	Gender (0=Male, 1=Female)	Simulated Patient (0=Not Deaf, 1=Deaf)	Region (1=Southwest, 2=North, 3=Southeast)	Density (0=Urban, 1=Rural)	Interpreter-related reason for unsuccessful attempt (0=No, 1=Yes, .=Missing)	Number of contacts
104	1	0	1	0	1	0		1
104	1	1	1	1	1	0	1	3
105	0	0	1	1	3	0		2
105	0	0	1	0	3	0		2
107	0	0	1	1	1	0		1
107	0	1	1	0	1	0	0	1
108	1	0	1	0	1	0		1
108	1	1	1	1	1	0	1	2
109	0	0	1	1	2	0		2
109	0	1	1	0	2	0	0	1
110	0	0	1	1	1	0		1
110	0	1	1	0	1	0	0	1
111	0	1	1	1	3	0	1	1
111	0	1	1	0	3	0	0	1
113	1	1	1	1	1	0	1	1
113	1	0	0	0	1	0		1
114	0	0	1	1	3	1		1
114	0	1	0	0	3	1	0	1
115	0	0	1	1	1	0		1
115	0	1	0	0	1	0	0	1
116	1	0	1	1	3	0		1
116	1	0	0	0	3	0		1
117	1	0	1	1	3	1		2
117	1	0	0	0	3	1		1
119	1	0	1	1	3	0		1

© 2021 Schniedewind E et al. *JAMA Network Open.*

Office ID	Clinic type (0=Primary Care, 1=Dental)	Appointment Success (0=Yes, 1=No)	Gender (0=Male, 1=Female)	Simulated Patient (0=Not Deaf, 1=Deaf)	Region (1=Southwest, 2=North, 3=Southeast)	Density (0=Urban, 1=Rural)	Interpreter-related reason for unsuccessful attempt (0=No, 1=Yes, .=Missing)	Number of contacts
119	1	0	0	0	3	0		1
121	1	1	1	1	1	0	0	3
121	1	0	0	0	1	0		1
124	0	0	0	0	1	0		1
124	0	0	1	1	1	0		1
128	0	1	0	0	2	0	0	1
128	0	0	1	1	2	0		1
129	0	1	0	0	2	0	0	1
129	0	1	1	1	2	0	0	1
131	0	0	1	1	3	0		1
131	0	0	0	0	3	0		1
132	0	0	0	0	3	1		1
132	0	0	1	1	3	1		1
136	1	0	0	0	1	0		1
136	1	0	1	1	1	0		1
137	0	1	0	0	1	0	0	1
137	0	1	1	1	1	0	0	1
138	0	0	1	1	1	0		1
138	0	0	0	0	1	0		1
140	1	0	0	0	1	0		1
140	1	1	1	1	1	0	0	1
141	0	0	1	1	2	1		1
141	0	0	0	0	2	1		1
142	1	0	0	0	2	1		1
142	1	1	1	1	2	1	1	1

© 2021 Schniedewind E et al. *JAMA Network Open.*

Office ID	Clinic type (0=Primary Care, 1=Dental)	Appointment Success (0=Yes, 1=No)	Gender (0=Male, 1=Female)	Simulated Patient (0=Not Deaf, 1=Deaf)	Region (1=Southwest, 2=North, 3=Southeast)	Density (0=Urban, 1=Rural)	Interpreter-related reason for unsuccessful attempt (0=No, 1=Yes, .=Missing)	Number of contacts
143	0	0	1	1	3	1		1
143	0	0	0	0	3	1		1
144	1	1	1	1	2	1	1	2
144	1	0	0	0	2	1		1
146	1	0	0	0	2	0		1
146	1	1	1	1	2	0	0	1
148	1	0	1	1	3	1		1
148	1	0	0	0	3	1		1
149	0		0	0	2	0	0	1
149	0	0	1	1	2	0		1
150	0	0	1	1	1	0		1
150	0	0	0	0	1	0		1
152	0	1	1	1	2	0	1	1
152	0	0	0	0	2	0		1
153	0	1	1	1	2	0	0	
153	0	0		0	2	0		
154	0	0	0	0	1	0		1
154	0	0	1	1	1	0		1
155	0		0	0	3	0		1
155	0	1	1	1	3	0	0	1
156	1	0	1	1	1	0		1
156	1	1	0	0	1	0	0	1
157	0	1	0	0	1	0	0	1
157	0	1	1	1	1	0	0	1
158	0	1	1	1	1	0	0	1

© 2021 Schniedewind E et al. *JAMA Network Open.*

Office ID	Clinic type (0=Primary Care, 1=Dental)	Appointment Success (0=Yes, 1=No)	Gender (0=Male, 1=Female)	Simulated Patient (0=Not Deaf, 1=Deaf)	Region (1=Southwest, 2=North, 3=Southeast)	Density (0=Urban, 1=Rural)	Interpreter-related reason for unsuccessful attempt (0=No, 1=Yes, .=Missing)	Number of contacts
158	0	1	0	0	1	0	0	1
159	0	1	1	1	1	0	1	1
159	0	0	0	0	1	0		1
161	0	1	0	0	3	1	0	2
161	0	0	1	1	3	1		1
163	0	1	1	1	1	1	0	1
163	0	1	0	0	1	1	0	1
164	0	0	1	1	1	0		1
164	0	1	0	0	1	0	0	1
165	0	0	0	0	1	0		1
165	0	0	1	1	1	0		1
166	0	0	0	0	1	0		2
166	0	1	1	1	1	0	0	1
167	0	1	1	1	3	0	0	1
167	0	1	0	0	3	0	0	1
168	1	0	0	0	2	0		1
168	1	1	1	1	2	0		1
170	1	1	1	1	3	1	0	1
170	1	0	0	0	3	1		1
171	0	1	1	1	1	0	0	1
171	0	0	0	0	1	0		1
172	0	1	0	0	1	0	0	1
172	0	1	1	1	1	0	0	1
173	0	1	0	0	1	0	0	1
173	0	1	1	1	1	0	0	1

© 2021 Schniedewind E et al. *JAMA Network Open.*

Office ID	Clinic type (0=Primary Care, 1=Dental)	Appointment Success (0=Yes, 1=No)	Gender (0=Male, 1=Female)	Simulated Patient (0=Not Deaf, 1=Deaf)	Region (1=Southwest, 2=North, 3=Southeast)	Density (0=Urban, 1=Rural)	Interpreter-related reason for unsuccessful attempt (0=No, 1=Yes, .=Missing)	Number of contacts
174	1	1	1	1	1	0	1	1
174	1	0	0	0	1	0		1
175	0	0	0	0	3	1		1
175	0	0	1	1	3	1		1
176	0	0	1	1	1	0		1
176	0	0	0	0	1	0		1
178	0	0	1	1	3	0		1
178	0	1	0	0	3	0	0	1
179	0	0	1	1	1	0		1
179	0	0	0	0	1	0		1
180	0	0	0	0	3	0		1
180	0	0	1	1	3	0		1
181	0	1	1	1	3	0	0	1
181	0	1	0	0	3	0	0	1
182	1	0	0	0	3	0		1
182	1	1	1	1	3	0	1	1
183	0	0	1	1	1	0		1
183	0	0	0	0	1	0		1
184	0	1	0	0	3	0	0	1
184	0	1	1	1	3	0	0	1
185	0	1	1	1	1	0	0	1
185	0	0	0	0	1	0		2
187	1	0	0	0	1	0		1
187	1	1	1	1	1	0	1	1
188	0	0	1	1	1	0		1

© 2021 Schniedewind E et al. *JAMA Network Open.*

Office ID	Clinic type (0=Primary Care, 1=Dental)	Appointment Success (0=Yes, 1=No)	Gender (0=Male, 1=Female)	Simulated Patient (0=Not Deaf, 1=Deaf)	Region (1=Southwest, 2=North, 3=Southeast)	Density (0=Urban, 1=Rural)	Interpreter-related reason for unsuccessful attempt (0=No, 1=Yes, .=Missing)	Number of contacts
188	0	0	0	0	1	0		1
189	0	0	1	1	2	1		1
189	0	0	0	0	2	1		1
190	0	0	0	0	2	1		1
190	0	0	1	1	2	1		1
192	0	1	0	0	1	1	0	1
192	0	1	1	1	1	1	0	1
193	0	0	1	1	3	1		1
193	0	0	0	0	3	1		1
195	1	0	0	0	1	0		1
195	1	1	1	1	1	0	1	1
198	0	1	1	1	1	1	0	1
198	0	0	0	0	1	1		1
199	0	1	1	1	3	1	0	1
199	0	0	0	0	3	1		1
203	0	1	0	0	2	0	0	1
203	0	1	1	1	2	0	1	1
204	1	0	0	0	1	0		1
204	1	0	1	1	1	0		1
206	0	0	0	0	3	1		1
206	0	1	1	1	3	1	1	1
207	1	0	0	0	2	1		1
207	1	1	1	1	2	1		1
209	0	0	1	1	1	0		1
209	0	1	0	0	1	0	0	1

© 2021 Schniedewind E et al. *JAMA Network Open.*

Office ID	Clinic type (0=Primary Care, 1=Dental)	Appointment Success (0=Yes, 1=No)	Gender (0=Male, 1=Female)	Simulated Patient (0=Not Deaf, 1=Deaf)	Region (1=Southwest, 2=North, 3=Southeast)	Density (0=Urban, 1=Rural)	Interpreter-related reason for unsuccessful attempt (0=No, 1=Yes, .=Missing)	Number of contacts
210	0	0	1	1	1	0		1
210	0	1	0	0	1	0	0	1
213	1	1	1	1	1	0	1	1
213	1	1	0	0	1	0	0	1
214	0	1	0	0	2	0	0	1
214	0	1	1	1	2	0	0	1
215	0	0	1	1	1	0		1
215	0	0	0	0	1	0		1
216	1	1	1	1	2	0	1	1
216	1	1	0	0	2	0	0	1
218	0	1	1	1	1	0	0	1
218	0	1	0	0	1	0	0	2
220	0	0	0	0	2	0		1
220	0	1	1	1	2	0	1	1
223	1	0	1	1	3	1		1
223	1	1	0	0	3	1	0	1
224	0	1	0	0	2	0	0	1
224	0	1	1	1	2	0	0	1
225	0	0	1	0	3	0		1
225	0	1	0	1	3	0	1	1
226	1	0	1	0	3	1		1
226	1	1	0	1	3	1	1	1
227	0	0	0	1	2	0		1
227	0	0	1	0	2	0		1
228	0	1	0	1	1	0	0	2

© 2021 Schniedewind E et al. *JAMA Network Open.*

Office ID	Clinic type (0=Primary Care, 1=Dental)	Appointment Success (0=Yes, 1=No)	Gender (0=Male, 1=Female)	Simulated Patient (0=Not Deaf, 1=Deaf)	Region (1=Southwest, 2=North, 3=Southeast)	Density (0=Urban, 1=Rural)	Interpreter-related reason for unsuccessful attempt (0=No, 1=Yes, .=Missing)	Number of contacts
228	0	0	1	0	1	0		1
229	0	1	1	0	3	0	0	1
229	0	1	0	1	3	0	0	1
230	0	0	0	1	1	0		1
230	0	0	1	0	1	0		1
231	0	0	0	1	2	0		1
231	0	1	1	0	2	0	0	1
232	1	0	1	0	3	1		1
232	1	1	0	1	3	1	1	1
233	0	1	1	0	3	0	0	1
233	0	0	0	1	3	0		4
234	0	0	1	0	3	1		1
234	0	1	0	1	3	1	0	1
235	0	0	1	0	1	0		1
235	0	0	0	1	1	0		1
241	0	1	1	0	1	1	0	1
241	0	1	0	1	1	1	0	1
242	1	0	1	0	2	0		1
242	1	1	0	1	2	0	1	2
243	1	0	1	0	2	0		1
243	1	1	0	1	2	0	0	2
244	0	1	1	0	1	0	0	1
244	0	1	0	1	1	0	0	1
245	1	1	0	1	1	0	0	1
245	1	1	1	0	1	0	0	1

© 2021 Schniedewind E et al. *JAMA Network Open.*

Office ID	Clinic type (0=Primary Care, 1=Dental)	Appointment Success (0=Yes, 1=No)	Gender (0=Male, 1=Female)	Simulated Patient (0=Not Deaf, 1=Deaf)	Region (1=Southwest, 2=North, 3=Southeast)	Density (0=Urban, 1=Rural)	Interpreter-related reason for unsuccessful attempt (0=No, 1=Yes, .=Missing)	Number of contacts
246	1	1	0	1	2	0	1	2
246	1	0	1	0	2	0		1
247	0	1	1	0	3	0	0	1
247	0	1	0	1	3	0	0	1
248	0	1	1	0	3	0	0	1
248	0	1	0	1	3	0	0	1
249	0	1	0	1	1	1	0	2
249	0	1	1	0	1	1	0	1
250	1	0	1	0	1	0		1
250	1	0	0	1	1	0		3
251	0	1	1	0	2	1	0	1
251	0	1	0	1	2	1	0	2
252	1	0	1	0	1	0		1
252	1	0	0	1	1	0		3
253	0	0	1	0	1	0		1
253	0	1	0	1	1	0	0	2
254	1	0	1	0	1	0		1
254	1	1	0	1	1	0	1	3
256	0	0	1	0	1	0		1
256	0	0	0	1	1	0		2
258	0	1	0	1	1	0	0	1
258	0	1	1	0	1	0	0	1
259	0	0	1	0	3	0		1
259	0	1	0	1	3	0	1	1
260	1	0	1	0	1	0		1

© 2021 Schniedewind E et al. *JAMA Network Open.*

Office ID	Clinic type (0=Primary Care, 1=Dental)	Appointment Success (0=Yes, 1=No)	Gender (0=Male, 1=Female)	Simulated Patient (0=Not Deaf, 1=Deaf)	Region (1=Southwest, 2=North, 3=Southeast)	Density (0=Urban, 1=Rural)	Interpreter-related reason for unsuccessful attempt (0=No, 1=Yes, .=Missing)	Number of contacts
260	1	1	0	1	1	0	1	2
261	1	0	0	1	3	0		2
261	1	0	1	0	3	0		1
262	1	1	0	1	1	0	1	3
262	1	0	1	0	1	0		1
264	0	0	1	0	3	1		1
264	0	1	0	1	3	1	1	1
265	0	1	0	1	3	1	1	2
265	0	0	1	0	3	1		1
267	0	1	1	0	3	1	0	1
267	0	0	0	1	3	1		2
268	0	1	1	0	1	0	0	1
268	0	0	0	1	1	0		1
274	1	0	0	1	2	0		3
274	1	1	1	0	2	0	0	1
275	0	0	0	1	1	0		1
275	0	1	1	0	1	0	0	1
276	0	1	1	0	3	0	0	1
276	0	0	0	1	3	0		3
278	0	1	1	0	3	1	0	1
278	0	1	0	1	3	1	1	1
280	1	0	1	0	3	1		1
280	1	1	0	1	3	1	1	1
281	1	0	0	1	2	1		2
281	1	1	1	0	2	1	0	1

© 2021 Schniedewind E et al. *JAMA Network Open.*

Office ID	Clinic type (0=Primary Care, 1=Dental)	Appointment Success (0=Yes, 1=No)	Gender (0=Male, 1=Female)	Simulated Patient (0=Not Deaf, 1=Deaf)	Region (1=Southwest, 2=North, 3=Southeast)	Density (0=Urban, 1=Rural)	Interpreter-related reason for unsuccessful attempt (0=No, 1=Yes, .=Missing)	Number of contacts
282	0	0	0	1	3	1		2
282	0	0	1	0	3	1		1
283	0	1	0	1	3	0	0	1
283	0	1	1	0	3	0	0	1
284	1	0	1	0	3	1		1
284	1	0	0	1	3	1		2
285	0	0	0	1	3	1		1
285	0	1	1	0	3	1	0	1
286	0	1	1	0	3	0	0	1
286	0	1	0	1	3	0	0	3
287	0	1	0	1	1	0	1	3
287	0	0	1	0	1	0		1
288	0	1	0	1	3	0	0	1
288	0	0	1	0	3	0		1
291	0	1	0	1	3	1	1	1
291	0	0	1	0	3	1		1
292	1	0	1	0	2	0		1
292	1	1	0	1	2	0	0	2
293	0	1	1	0	1	0	0	1
293	0	0	0	1	1	0		1
294	0	0	1	0	3	1		1
294	0	1	0	1	3	1	0	2
296	0	0	1	0	1	1		1
296	0	1	0	1	1	1	0	1
297	1	1	0	1	2	0	1	2

© 2021 Schniedewind E et al. *JAMA Network Open.*

Office ID	Clinic type (0=Primary Care, 1=Dental)	Appointment Success (0=Yes, 1=No)	Gender (0=Male, 1=Female)	Simulated Patient (0=Not Deaf, 1=Deaf)	Region (1=Southwest, 2=North, 3=Southeast)	Density (0=Urban, 1=Rural)	Interpreter-related reason for unsuccessful attempt (0=No, 1=Yes, .=Missing)	Number of contacts
297	1	0	1	0	2	0		1
298	0	1	1	0	2	0	0	1
298	0	0	0	1	2	0		1
299	0	1	0	1	3	0	1	2
299	0	1	1	0	3	0	0	1
300	0		1	0	1	0	0	1
300	0	0	0	1	1	0		1
302	0	0	0	1	1	0		1
302	0	0	1	0	1	0		1
303	1	0	1	0	1	0		1
303	1	0	0	1	1	0		2
306	0	1	1	0	2	0	0	1
306	0	1	0	1	2	0	0	2
307	1	0	0	1	1	0		1
307	1	1	1	0	1	0	0	1
308	0	1	1	0	3	1	0	1
308	0	0	0	1	3	1		1
310	0	1	0	1	1	0	0	1
310	0	1	1	0	1	0	0	1
311	0	1	1	0	1	0	0	1
311	0	1	0	1	1	0	0	1
314	0	1	0	1	3	0	0	1
314	0	1	1	0	3	0	0	1
315	0	0	0	1	1	0		1
315	0	1	1	0	1	0	0	1

© 2021 Schniedewind E et al. *JAMA Network Open.*

Office ID	Clinic type (0=Primary Care, 1=Dental)	Appointment Success (0=Yes, 1=No)	Gender (0=Male, 1=Female)	Simulated Patient (0=Not Deaf, 1=Deaf)	Region (1=Southwest, 2=North, 3=Southeast)	Density (0=Urban, 1=Rural)	Interpreter-related reason for unsuccessful attempt (0=No, 1=Yes, .=Missing)	Number of contacts
316	0	1	1	0	2	0	0	1
316	0	1	0	1	2	0	0	1
319	1	1	0	1	2	0	0	1
319	1	0	1	0	2	0		1
320	1	0	1	0	3	0		1
320	1	0	0	1	3	0		4
321	0	1	1	0	3	0	0	1
321	0	1	0	1	3	0	1	2
322	0	0	1	0	3	0		1
322	0	0	0	1	3	0		2
323	1	1	1	0	1	0	0	1
323	1	1	0	1	1	0	1	2
324	1	0	0	1	3	0		3
324	1	0	1	0	3	0		1
326	0	1	1	0	3	1	0	1
326	0	1	0	1	3	1	0	1
331	1	1	0	1	3	1	1	3
331	1	0	1	0	3	1		1
334	0	1	0	1	3	0	1	4
334	0	0	1	0	3	0		1
335	0	1	1	0	1	0	0	1
335	0	1	0	1	1	0	1	2
336	0	0	1	0	1	0		1
336	0	0	0	1	1	0		2
339	0	1	0	1	2	0	0	1

© 2021 Schniedewind E et al. *JAMA Network Open.*

Office ID	Clinic type (0=Primary Care, 1=Dental)	Appointment Success (0=Yes, 1=No)	Gender (0=Male, 1=Female)	Simulated Patient (0=Not Deaf, 1=Deaf)	Region (1=Southwest, 2=North, 3=Southeast)	Density (0=Urban, 1=Rural)	Interpreter-related reason for unsuccessful attempt (0=No, 1=Yes, .=Missing)	Number of contacts
339	0	1	0	0	2	0	0	1
340	0	0	0	0	2	0		1
340	0	0	0	1	2	0		1
342	0	0	0	1	1	1		1
342	0	0	0	0	1	1		1
344	0	0	0	0	3	0		1
344	0	0	0	1	3	0		1
345	0	0	0	1	1	0		1
345	0	0	0	0	1	0		1
346	0	0	0	1	3	1		2
346	0	0	0	0	3	1		1
347	0	0	0	0	3	0		1
347	0	0	0	1	3	0		1
348	0	1	0	1	1	0	0	1
348	0	1	0	0	1	0	0	1
349	0	0	0	0	1	0		1
349	0	1	0	1	1	0	1	1
350	0	1	0	1	1	0	0	2
350	0	1	0	0	1	0	0	1
351	0	1	0	1	3	0	0	1
351	0	1	0	0	3	0	0	1
352	0	1	0	0	2	0	0	1
352	0	1	0	1	2	0	0	1
353	0	1	0	0	1	0	0	1
353	0	0	0	1	1	0		1

© 2021 Schniedewind E et al. *JAMA Network Open.*

Office ID	Clinic type (0=Primary Care, 1=Dental)	Appointment Success (0=Yes, 1=No)	Gender (0=Male, 1=Female)	Simulated Patient (0=Not Deaf, 1=Deaf)	Region (1=Southwest, 2=North, 3=Southeast)	Density (0=Urban, 1=Rural)	Interpreter-related reason for unsuccessful attempt (0=No, 1=Yes, .=Missing)	Number of contacts
356	0	0	0	1	3	1		2
356	0	0	0	0	3	1		1
359	1	0	0	0	3	0		1
359	1	0	0	1	3	0		2
360	0	0	0	0	3	0		1
360	0	1	0	1	3	0	0	1
361	0	1	0	1	3	0	0	1
361	0	1	0	0	3	0	0	1
363	0	1	0	1	1	0	0	1
363	0	1	0	0	1	0	0	1
364	1	0	0	0	2	0		1
364	1	1	0	1	2	0	1	4
365	0	1	0	0	1	0	0	1
365	0	1	0	1	1	0	0	1
366	0	0	0	0	2	1		1
366	0	1	0	1	2	1	1	2
368	1	0	0	1	1	1		3
368	1	0	0	0	1	1		1
370	0	1	0	0	2	0	0	1
370	0	0	0	1	2	0		1
371	1	0	0	0	3	1		1
371	1	0	0	1	3	1		1
374	1	1	0	1	3	0	1	3
374	1	0	0	0	3	0		1
375	0	0	0	1	2	0		2

© 2021 Schniedewind E et al. *JAMA Network Open.*

Office ID	Clinic type (0=Primary Care, 1=Dental)	Appointment Success (0=Yes, 1=No)	Gender (0=Male, 1=Female)	Simulated Patient (0=Not Deaf, 1=Deaf)	Region (1=Southwest, 2=North, 3=Southeast)	Density (0=Urban, 1=Rural)	Interpreter-related reason for unsuccessful attempt (0=No, 1=Yes, .=Missing)	Number of contacts
375	0	0	0	0	2	0		1
376	1	0	0	0	3	0		1
376	1	0	0	1	3	0		2
377	0	0	0	1	1	0		1
377	0	0	0	0	1	0		1
378	0	0	0	1	3	0		1
378	0	1	0	0	3	0	0	1
379	1	0	0	1	2	0		3
379	1	0	0	0	2	0		1
380	1	0	0	0	3	1		1
380	1	0	0	1	3	1		2
382	1	0	0	1	1	0		1
382	1	0	0	0	1	0		1
385	0	0	0	1	1	0		3
385	0	0	0	0	1	0		1
386	1	0	0	1	1	1		4
386	1	0	0	0	1	1		1
387	0	0	0	0	2	0		1
387	0	1	0	1	2	0	0	1
388	1	0	0	0	1	0		1
388	1	1	0	1	1	0	1	1
391	0	0	0	1	2	0		3
391	0	0	0	0	2	0		1
392	0	0	0	1	1	1		1
392	0	0	0	0	1	1		1

© 2021 Schniedewind E et al. *JAMA Network Open.*

Office ID	Clinic type (0=Primary Care, 1=Dental)	Appointment Success (0=Yes, 1=No)	Gender (0=Male, 1=Female)	Simulated Patient (0=Not Deaf, 1=Deaf)	Region (1=Southwest, 2=North, 3=Southeast)	Density (0=Urban, 1=Rural)	Interpreter-related reason for unsuccessful attempt (0=No, 1=Yes, .=Missing)	Number of contacts
393	0	0	0	1	3	1		1
393	0	0	0	0	3	1		1
394	1	0	0	1	1	0		1
394	1	0	0	0	1	0		1
396	1	0	0	1	2	1		5
396	1	0	0	0	2	1		1
397	0	0	0	0	3	1		1
397	0	1	0	1	3	1	1	2
398	0	1	0	0	1	0	0	1
398	0	0	0	1	1	0		1
399	0	0	0	0	3	1		1
399	0	0	0	1	3	1		1
400	0	0	0	0	3	1		1
400	0	1	0	1	3	1	1	1
401	0	0	0	0	1	0		1
401	0	1	0	1	1	0	1	1
402	1	1	0	1	1	0	1	1
402	1	0	0	0	1	0		1
403	1	1	0	0	1	0	0	1
403	1	1	0	1	1	0	1	1
404	0	1	0	1	1	0	0	1
404	0	0	0	0	1	0		1
405	0	0	0	0	2	0		1
405	0	0	0	1	2	0		1
406	1	1	0	0	3	0	0	1

© 2021 Schniedewind E et al. *JAMA Network Open.*

Office ID	Clinic type (0=Primary Care, 1=Dental)	Appointment Success (0=Yes, 1=No)	Gender (0=Male, 1=Female)	Simulated Patient (0=Not Deaf, 1=Deaf)	Region (1=Southwest, 2=North, 3=Southeast)	Density (0=Urban, 1=Rural)	Interpreter-related reason for unsuccessful attempt (0=No, 1=Yes, .=Missing)	Number of contacts
406	1	0	0	1	3	0		3
407	1	0	0	0	3	0		1
407	1	0	0	1	3	0		2
408	0	0	0	1	2	0		1
408	0	0	0	0	2	0		1
409	0	0	0	0	2	0		1
409	0	0	0	1	2	0		2
411	0	1	0	0	1	0	0	1
411	0	0	0	1	1	0		1
412	1	0	0	1	1	0		1
412	1	0	0	0	1	0		1
413	1	0	0	0	3	1		1
413	1	1	0	1	3	1	1	1
414	0	1	0	1	3	0	0	1
414	0	0	0	0	3	0		1
415	0	0	0	1	3	0		2
415	0	1	0	0	3	0	0	1
416	1	1	0	1	2	1	0	1
416	1	1	0	0	2	1	0	1
417	0	0	0	1	2	0		2
417	0	1	0	0	2	0	0	1
418	0	0	0	0	3	0		1
418	0	0	0	1	3	0		1
419	0	0	0	0	2	0		1
419	0	0	0	1	2	0		1

© 2021 Schniedewind E et al. *JAMA Network Open.*

Office ID	Clinic type (0=Primary Care, 1=Dental)	Appointment Success (0=Yes, 1=No)	Gender (0=Male, 1=Female)	Simulated Patient (0=Not Deaf, 1=Deaf)	Region (1=Southwest, 2=North, 3=Southeast)	Density (0=Urban, 1=Rural)	Interpreter-related reason for unsuccessful attempt (0=No, 1=Yes, .=Missing)	Number of contacts
420	0	0	0	1	2	0		1
420	0	0	0	0	2	0		1
422	0	0	0	1	1	0		3
422	0	0	0	0	1	0		1
423	0	0	0	0	1	1		1
423	0	0	0	1	1	1		2
424	0	0	0	0	1	0		1
424	0	0	0	1	1	0		2
427	0	0	0	1	2	1		3
427	0	0	0	0	2	1		1
428	1	1	0	1	3	0	1	3
428	1	0	0	0	3	0		1
429	1	0	0	1	2	1		3
429	1	1	0	0	2	1	0	1
430	1	1	0	1	1	0	1	1
430	1	0	0	0	1	0		1
431	0	0	0	1	1	0		
431	0	0	0	0	1	0		
435	0	0	0	0	1	0		1
435	0	1	0	1	1	0	1	1
436	0	1	0	0	1	0	0	1
436	0	1	0	1	1	0	0	1
437	0	1	0	1	1	0	0	1
437	0	0	0	0	1	0		1
438	0	1	0	1	2	1	0	1

© 2021 Schniedewind E et al. *JAMA Network Open.*

Office ID	Clinic type (0=Primary Care, 1=Dental)	Appointment Success (0=Yes, 1=No)	Gender (0=Male, 1=Female)	Simulated Patient (0=Not Deaf, 1=Deaf)	Region (1=Southwest, 2=North, 3=Southeast)	Density (0=Urban, 1=Rural)	Interpreter-related reason for unsuccessful attempt (0=No, 1=Yes, .=Missing)	Number of contacts
438	0	1	0	0	2	1	0	1
439	0	1	0	0	1	0	0	1
439	0	1	0	1	1	0	0	1
440	1	1	0	1	1	0	0	1
440	1	0	0	0	1	0		1
441	1	0	0	0	1	0		1
441	1	1	0	1	1	0	0	3
442	0	1	0	1	3	1	0	1
442	0	1	0	0	3	1	0	1
444	1	1	0	0	3	1	0	1
444	1	1	0	1	3	1	1	1

© 2021 Schniedewind E et al. *JAMA Network Open.*
